# Effects of phospho- and calciotropic hormones on electrolyte transport in the proximal tubule

**DOI:** 10.12688/f1000research.12097.1

**Published:** 2017-10-03

**Authors:** Justin J. Lee, Allein Plain, Megan R. Beggs, Henrik Dimke, R. Todd Alexander

**Affiliations:** 1 Department of Physiology, University of Alberta, Edmonton, Canada; 2 The Women and Children’s Health Research Institute, Edmonton, Canada; 3 Department of Cardiovascular and Renal Research, Institute of Molecular Medicine, University of Southern Denmark, Odense, Denmark; 4 Department of Pediatrics, Edmonton Clinic Health Academy, University of Alberta, Edmonton, Canada

**Keywords:** Parathyroid hormone, fibroblast growth factor 23, phosphotropic hormomes, calciotropic hormones

## Abstract

Calcium and phosphate are critical for a myriad of physiological and cellular processes within the organism. Consequently, plasma levels of calcium and phosphate are tightly regulated. This occurs through the combined effects of the phospho- and calciotropic hormones, parathyroid hormone (PTH), active vitamin D _3_, and fibroblast growth factor 23 (FGF23). The organs central to this are the kidneys, intestine, and bone. In the kidney, the proximal tubule reabsorbs the majority of filtered calcium and phosphate, which amounts to more than 60% and 90%, respectively. The basic molecular mechanisms responsible for phosphate reclamation are well described, and emerging work is delineating the molecular identity of the paracellular shunt wherein calcium permeates the proximal tubular epithelium. Significant experimental work has delineated the molecular effects of PTH and FGF23 on these processes as well as their regulation of active vitamin D _3_ synthesis in this nephron segment. The integrative effects of both phospho- and calciotropic hormones on proximal tubular solute transport and subsequently whole body calcium-phosphate balance thus have been further complicated. Here, we first review the molecular mechanisms of calcium and phosphate reabsorption from the proximal tubule and how they are influenced by the phospho- and calciotropic hormones acting on this segment and then consider the implications on both renal calcium and phosphate handling as well as whole body mineral balance.

## Introduction

The kidneys play a critical role in maintaining electrolyte balance, including both calcium and phosphate. They accomplish this by adjusting the urinary excretion of these minerals, thereby amending the amount in blood. In particular, the proximal tubule (PT) reabsorbs approximately 70% of filtered calcium and 90% of filtered phosphate ions ^1^. Failure to properly regulate PT reabsorption leads to abnormal calcium and phosphate homeostasis, which may manifest as neuromuscular, cardiovascular, or gastrointestinal symptoms ^2^. The molecular mechanisms mediating calcium and phosphate reabsorption in the PT, as well as the endocrine regulation of these processes, have been extensively studied. The endocrine factors involved in calcium and phosphate homeostasis are known as calciotropic and phosphotropic hormones, respectively. These hormones include parathyroid hormone (PTH), 1,25-dihydroxyvitamin D _3_ (that is, active vitamin D), and fibroblast growth factor 23 (FGF23). Previous review articles have primarily focused on the individual effects of PTH, active vitamin D, and FGF23 on either calcium or phosphate transport in the kidneys, and some have suggested an interconnection between the two pathways ^1, 3– 6^. However, emerging work demonstrates that PTH and FGF23 each have distinct effects on both phosphate and calcium homeostasis. They should thus be considered calciophosphotropic hormones, a term we will use for the remainder of this article. Here, we briefly review calcium and phosphate reabsorption and their dependence on sodium transport in the PT and then dissect the role of PTH and FGF23 on these processes.

## Proximal tubule

The PT is the initial segment of the nephron wherein transport occurs. It is responsible for reabsorbing the majority of water and solutes that filter into this tubular segment from the glomerulus. Anatomically, the PT is located in the renal cortex and can be divided into (i) the proximal convoluted tubule (PCT) and (ii) the proximal straight tubule (PST). The PT can be further subdivided into segments S1, S2, and S3 on the basis of molecular ultrastructure and expression profiles ^7– 10^. The PCT is comprised of S1 and part of S2, whereas the PST contains the remainder of the S2 segment as well as the S3 segment ^7, 8^. The majority of sodium, bicarbonate, and phosphate reabsorption from the PT occurs in the PCT, owing to the greater expression of select sodium-coupled cotransporters, larger microvilli surface area, and denser mitochondrial population. In contrast, calcium reabsorption occurs in the distal part of the PT due to a favourable electrochemical gradient there.

### Proximal tubular function

The transport of solutes across the PT epithelium occurs via both transcellular and paracellular pathways. The transcellular pathway is generally a unidirectional, active process whereby substrates that are reabsorbed in the PT enter the epithelial cell across the apical membrane and subsequently are extruded across the basolateral membrane. The paracellular pathway in the PT is either a passive or secondarily active bidirectional process permitted by tight-junction proteins called claudins ^11^. Transport via the paracellular pathway is determined by the transepithelial electrochemical gradient and the permeability of the tight junction. About 65% of transepithelial sodium reabsorption in the nephron occurs in the PT, and two thirds of it occurs via the transcellular pathway in a process coupled to bicarbonate reclamation ^12, 13^. As such, significant paracellular sodium reabsorption also takes place in this segment. Calcium reabsorption from the PT is primarily mediated by the paracellular pathway, while phosphate reabsorption occurs via the transcellular pathway ^14, 15^. Both calcium and phosphate reabsorption in the PT are dependent to some degree on the transepithelial transport of sodium.

### The proximal tubule, a target of calciophosphotropic hormones

**Figure 1.**
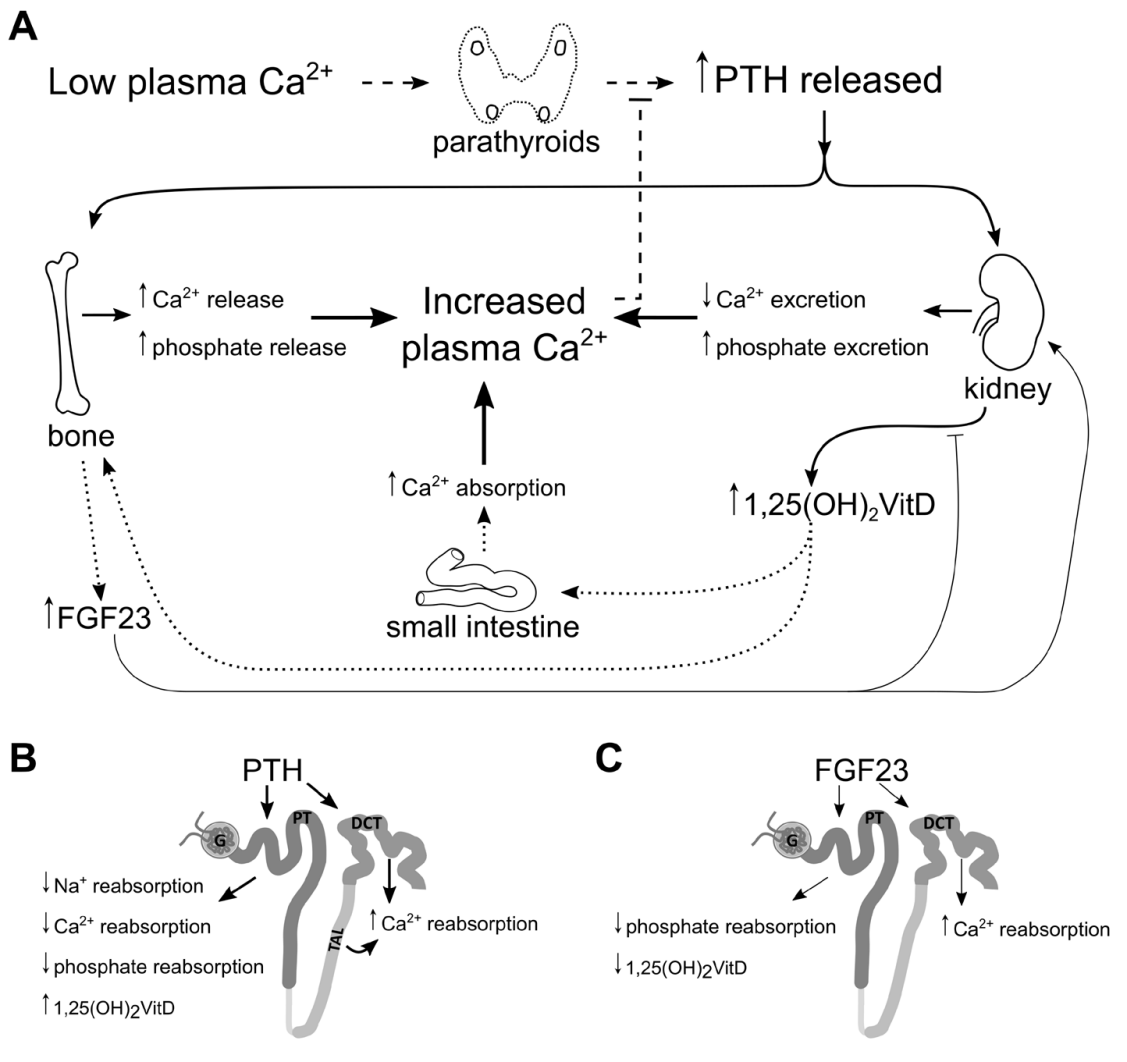
Regulation of calcium and phosphate by parathyroid hormone (PTH), 1,25-dihydroxyvitamin D _3_ (active vitamin D), and fibroblast growth factor 23 (FGF23). ( **A**) Low plasma calcium stimulates release of PTH from the parathyroid glands. PTH stimulates resorption of bone, releasing calcium and phosphate into the plasma. In the kidney, PTH increases urinary calcium reabsorption and phosphate excretion. ( **B**) PTH-dependent active calcium reabsorption takes place in the distal nephron but, in the proximal tubule (PT), stimulates 1α-hydroxylase to convert 25-hydroxyvitamin D _3_ into active vitamin D and reduces the reabsorption of sodium, calcium, and phosphate. Active vitamin D increases calcium absorption from the small intestine and stimulates FGF23 secretion from bone. ( **C**) FGF23 acts as a negative feedback modulator of activated vitamin D activation and increases distal nephron calcium reabsorption while decreasing phosphate reabsorption from PT.

Electrolyte transport in the PT is regulated by multiple factors, including the calciophosphotropic hormones PTH and FGF23. These hormones interdependently regulate one another through the PTH-active vitamin D–FGF23 axes ( Figure 1) ^16^. The regulatory mechanisms within these axes are complex and beyond the scope of this review. (The reader is referred to several recent reviews covering this topic ^16– 19^.) Here, we focus on the effects of PTH, active vitamin D, and FGF23 on calcium and phosphate transport processes in the PT.

PTH is produced in the parathyroid gland and released when systemic calcium levels are reduced below the physiological set point. PTH increases serum calcium levels by directly increasing calcium resorption from bone and reabsorption from kidneys, while it indirectly stimulates intestinal absorption by increasing the synthesis of active vitamin D in the kidneys ^20– 23^. Concomitantly, PTH inhibits phosphate reabsorption in the PT, thereby increasing phosphate excretion into urine ^24^. These actions on the PT are mediated by its direct interaction with the G protein–coupled type 1 PTH receptors (PTHRs) expressed on both apical and basolateral membranes ^25^. The major effects of PTH binding to the PTHR in the PT are mediated by protein kinase A (PKA) and protein kinase C (PKC). These protein kinases are stimulated by the G _s_- and G _q/11_-protein pathways, respectively ^26^. It is noteworthy that the apical PTHR preferentially signals through the PKC pathway ^25^. Ultimately, these signalling pathways modulate both the expression and membrane localization of transport proteins involved in the reabsorption of sodium, calcium, and phosphate across the PT epithelium.

PTH also increases plasma levels of active vitamin D via a direct effect on PT epithelial cells ^17^. The enzyme responsible for hydroxylating 25-hydroxyvitamin D _3_ at the 1α-position, CYP27B1 or 1α-hydroxylase, is expressed in the PT and is upregulated by PTH ^27^. Though traditionally thought of as a calciotropic hormone, active vitamin D is a phospho- and calciotropic hormone that increases both serum calcium and phosphate levels by stimulating their intestinal absorption ^21^. It also suppresses the release of PTH by downregulating *PTH* gene expression and increasing calcium-sensing receptor (CaSR) expression in the parathyroid gland ^28, 29^. Even though active vitamin D directly increases the expression of proteins involved in calcium reabsorption in the distal nephron, it is unclear whether it exerts a direct effect on electrolyte handling in the PT which is independent of its secondary effects on other hormones.

FGF23 is a 251–amino acid peptide hormone synthesized and released from osteocytes and osteoblasts in response to elevations in systemic active vitamin D or phosphate or both ^16, 19^. The primary action of FGF23 is to reduce PT phosphate reabsorption via binding to specific FGF receptors (FGFRs), including 1, 3, and 4, which are expressed on the basolateral membrane throughout the PT ^30, 31^. Downstream signalling after FGFR activation reduces phosphate transporter expression and apical membrane localization in the PT. This signalling also depends on its cofactor, klotho, to activate the downstream signalling pathways. Though primarily thought of as a phosphotropic hormone, FGF23 is also a calciotropic hormone. With klotho as its cofactor, FGF23 directly modulates calcium reabsorption from the distal convoluted tubule (DCT) ^32, 33^. Of note, PTH stimulates FGF23 release in rodents ^29, 34, 35^, while in contrast to PTH, FGF23 indirectly suppresses the 1-hydroxylation of 25-dihydroxyvitamin D _3_^17^. Thus, like PTH and active vitamin D, FGF23 regulates both calcium and phosphate homeostasis and therefore can be considered a calciophosphotropic hormone ^6, 36^.

## Calcium reabsorption from the proximal tubule

The kidney efficiently reabsorbs 98–99% of filtered calcium ions. More than 60% of this reabsorption occurs in the PT, which is largely driven by diffusion through the paracellular shunt ^37– 39^. Micropuncture studies in mammals show a parallel relationship between PT calcium reabsorption and sodium reabsorption, which does not dissociate under a variety of circumstances, including the administration of PTH, acetazolamide, furosemide, or hydrochlorothiazide or with the induction of acute and chronic metabolic acidosis ^40– 43^. In addition, an active transcellular pathway is proposed to account for less than 20% of calcium reabsorption from this segment ^37, 44^. Consistent with this, microperfusion experiments performed in the absence of a transepithelial potential difference found that not all calcium transport in the distal PT was passive and paracellular ^14^. Moreover, similar studies in the PST (S2 and S3 regions) of rabbit kidney demonstrate significant calcium transport that is independent of sodium transport, implying the presence of a transcellular calcium reabsorption pathway in the later portion of the PT ^45, 46^. The molecular constituents of this pathway remain to be elucidated. Towards this goal, an *in vitro* study using the L-type calcium channel blocker, nifedipine, abolished calcium flux in a rabbit PT cell model, implying the presence of functional apical L-type calcium channels in the PT ^47^. In addition, cation-permeable transient receptor potential channel 1 (TRPC1) has been localized to the apical membrane of PT cells *in vitro* and *in vivo* ^47, 48^. These studies support the presence of a transcellular pathway for calcium reabsorption in the PST; however, further study is required to delineate the molecular constituents.

### Calcium transport in the proximal tubule is coupled to sodium and water transport

**Figure 2.**
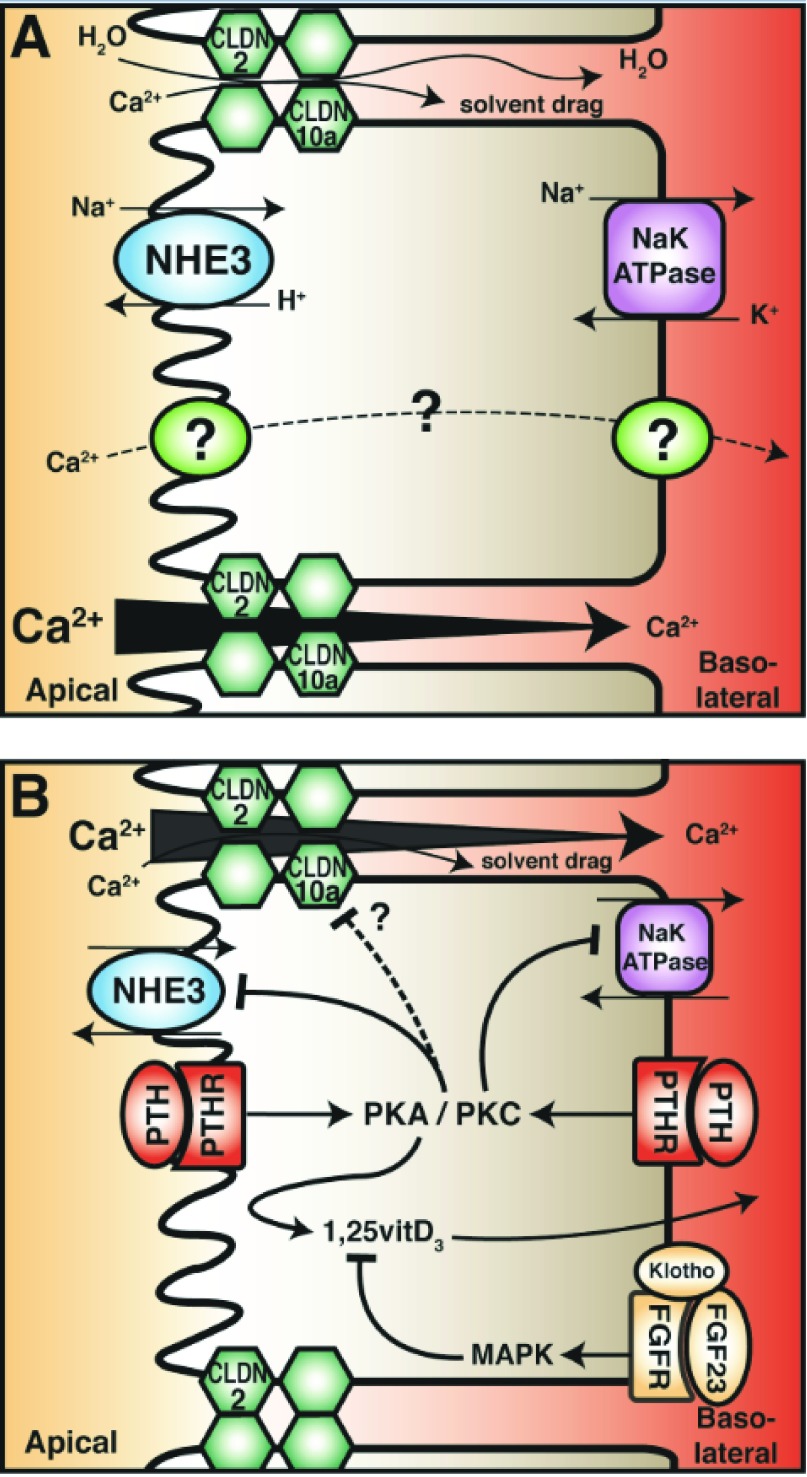
Proximal tubule (PT) calcium (Ca ^2+^) reabsorption. ( **A**) Calcium reabsorption from the PT occurs primarily by a paracellular route, likely mediated by claudin-2 (CLDN2). This is dependent on transcellular sodium reabsorption, driven by the sodium proton exchanger (NHE3) and sodium potassium ATPases. The reabsorption of sodium generates an osmotic gradient for water reabsorption, which in turn drags other solutes (including calcium) in a process known as solvent drag (top junction). In the late PT, the calcium concentration gradient favours reabsorption (from apical to basolateral) as the majority of sodium and water reabsorption occurs in the early PT (bottom junction). The transcellular calcium reabsorption pathway, present in late PT, is illustrated as a dashed line. ( **B**) Parathyroid hormone (PTH) in the PT decreases calcium reabsorption by attenuating its driving force. PTH in both the tubular fluid and the blood binds its receptor (PTHR), which is expressed on both apical and basolateral membranes. This activates the downstream messengers protein kinase A (PKA) and protein kinase C (PKC). Note that apical PTHR preferentially activates PKC. Both pathways inhibit NHE3 activity and reduce abundance, but only PKC inhibits Na ^+^/K ^+^ ATPase activity. PTH also reduces tight-junction permeability in the PT and enhances active vitamin D _3_ synthesis. In contrast, fibroblast growth factor 23 (FGF23) reduces active vitamin D _3_ levels.

Calcium reabsorption in the PT is highly dependent on sodium transport. The kidneys filter more than 500 g of sodium and 180 L of water daily, while approximately 4 g of filtered sodium and 1–2 L of water is excreted in the urine ^49^. The PT reabsorbs about two thirds of the filtered sodium and water. Sodium reabsorption in the PT is primarily mediated by an active transcellular pathway ( Figure 2A) ^50^. Active reabsorption of sodium creates a small, albeit significant, osmotic gradient for water, which is reabsorbed trans- and paracellularly through the water-selective channel aquaporin-1 and tight-junction pore claudin-2, respectively ^51^. The majority of sodium transport across the apical membrane occurs via the sodium proton exchanger isoform 3 (NHE3), encoded by the *Slc9a3* gene, which is expressed along the PCT. Animals with a targeted deletion of *Slc9a3* have a significant reduction in sodium and water reabsorption from the PT and display hypotension ^13, 52– 54^. Though contributing minimally to sodium reabsorption from the PT, other apical membrane sodium-coupled cotransporters, including sodium-glucose, sodium-phosphate, and sodium–amino acid cotransporters, are expressed in this segment. These transporters account for less than 5% of total transcellular sodium reabsorption in the PT; thus, only those involved in phosphate transport will be discussed here.

Sodium is also reabsorbed from the PT through the paracellular pathway. The PT is very leaky, displaying a transepithelial resistance (TER) of 5–7 Ω·cm ^2^ ^11^. This leakiness is conferred by a tight-junction family of proteins called claudins. Claudin-2, -10a, and -17 are expressed in this nephron segment ^11, 55, 56^. Claudin-2 forms a cation-selective, water-permeable pore, permitting paracellular diffusion of sodium, calcium, and water down their electrochemical gradients while restricting the diffusion of larger macromolecules ^57^. In the early PCT (S1), the transepithelial potential difference is lumen-negative, generated by the electrogenic sodium-glucose cotransporter (SGLT2) ^58^. Although this electrogenically favours paracellular cation secretion, this is overcome by the large amount of active sodium and consequent water reabsorption described above. The movement of water across the PT, when it occurs through the paracellular pore, can in turn carry other ions, including calcium, even against their respective transepithelial electrochemical gradient, in a process known as solvent drag ( Figure 2). This is supported by experiments using pharmacological blockade of NHE3 in a PT cell model and NHE3 knockout mice that display increased urinary calcium excretion and reduced calcium transport across intestinal epithelia ^59– 61^. In contrast, the transepithelial potential difference across the late PT is lumen-positive, which is the result of reclamation of chloride ions and bicarbonate ^58^. This electrochemical gradient thus favours the reabsorption of sodium and calcium ^58^. As sodium and water are reabsorbed in the earlier portions of the PT, calcium in the tubular fluid becomes concentrated, generating a transepithelial chemical gradient that favours paracellular calcium reabsorption ^37, 40, 41^. These mechanisms in concert generate a transepithelial electrochemical gradient from lumen-to-blood that drives calcium reabsorption. Taken together, the majority of PT calcium reabsorption occurs via a paracellular pathway.

### Regulation of sodium and calcium transport in the proximal tubule

PTH increases serum calcium in part by increasing the reabsorption of filtered calcium from the renal tubule, thereby reducing calcium excretion into urine. Paradoxically, micropuncture studies in the dog revealed that PTH reduces sodium, fluid, and calcium reabsorption from the PT, even though it still lowered urinary calcium excretion ^43, 62^. This observation is reconciled by findings of enhanced calcium reabsorption in the later segments of the tubule following PTH administration ^62– 64^. Microperfusion of rabbit cortical thick ascending limb (TAL) in the presence of PTH led to an almost fivefold increase in calcium flux across the segment with similar findings in mice ^65, 66^. PTH also affects calcium reabsorption through a transcellular calcium transport pathway in the distal convolution, which relies on the calcium-permeable transient receptor potential V5 channel (TRPV5) ^67– 69^. Consistent with this, PTH activates TRPV5 by increasing the open probability of the channel, membrane abundance, and total expression ^68– 70^. A secondary effect of PTH on the DCT is to increase the amount of TRPV5 indirectly by increasing circulating active vitamin D levels, as this hormone also enhances calcium reclamation in the DCT ^71, 72^.

A number of studies on animals demonstrate that the major effect of PTH on the PT is to inhibit sodium reabsorption, resulting in a natriuresis ( Figure 2B) ^42, 43, 62, 73^. Various *in vitro* expression studies using opossum kidney (OK) cells found that acute and chronic incubation with PTH downregulates *Slc9a3* at the transcriptional level, which is abolished by PKA inhibition (a downstream effector of PTH binding its receptor PTHR) ^74– 76^. These studies also revealed that PTH decreases NHE3 membrane abundance ^75, 77^. These cell culture studies are supported by *in vivo* work on PTH-infused rats that display significantly reduced renal NHE3 expression ^78– 80^. Furthermore, microperfusion of rat PT after chronic PTH exposure found reduced transepithelial sodium transport along with increased sodium and water excretion ^78, 79^. Thus, PTH directly inhibits NHE3-dependent transport. The actions of PTH on NHE3 are mediated by the phosphorylation of NHE3 at residue Ser605 by PKA ^81^. Although PKC activation results in NHE3 inhibition, this effect is mediated through an unknown mechanism that does not appear to require direct NHE3 phosphorylation ^82^. Both PKA and PKC are thought to interact with NHE3 through its PDZ domain–containing linker protein, the sodium-hydrogen exchanger regulatory factor 1 (NHERF-1); however, the precise mode of interaction is incompletely understood ^83, 84^. Furthermore, the molecular details of PTH-mediated transcriptional regulation have not been fully elucidated ^74^.

PTH also inhibits sodium/potassium ATPase activity in the PT, which would secondarily inhibit the apical sodium-dependent cotransporter fundamental to transcellular sodium reabsorption ( Figure 2B). This occurs through the activation of PKC via a G _q/11_ protein–coupled pathway after PTHR binding ^85– 87^. Activated PKC translocates to the basolateral membrane and phosphorylates the alpha subunit of the sodium/potassium ATPase, inhibiting its activity ^88, 89^. Given the abovementioned role of NHE3 and the sodium/potassium ATPase in paracellular calcium reabsorption, their inhibition by PTH would inhibit sodium reabsorption from the PT, which would decrease paracellular calcium reabsorption. This seems in direct contrast to the primary role of PTH to increase serum calcium levels. The reasons for this remain unclear. A previous attempt to reconcile this observation suggested that NHE3 inhibition alkalinizes the tubular fluid via reduced hydrogen secretion, thereby reducing reabsorption of bicarbonate ^76, 78^. This hypothesis is supported by a micropuncture study, where acute administration of PTH increased distal delivery of bicarbonate, leading to an alkaline urine ^90^. Since TRPV5, which is expressed in the distal nephron, is activated by alkaline pH, this could increase distal transcellular calcium reabsorption through TRPV5 ^76, 78, 91^. Micropuncture data further demonstrate the uncoupling of sodium and calcium transport in the distal nephron ^42, 43, 62^. However, direct *in vivo* measurements of tubular pH after PTH administration and the consequent effect on calcium reabsorption in the distal tubule have not been made.

Alternatively, PTH-mediated inhibition of PT sodium reabsorption might affect urinary calcium excretion by altering the glomerular filtration rate (GFR). Multiple *in vivo* studies found that exogenous PTH administration decreases GFR ^92– 94^. Consistent with this, patients with primary hyperparathyroidism show significantly reduced GFR ^95^. An explanation for this observation is that PTH stimulates tubuloglomerular feedback by increasing the distal delivery of chloride. The majority of chloride reclamation from the PT occurs through the paracellular pathway, driven by the transepithelial electrochemical gradient ^96^. PTH-mediated inhibition of sodium reabsorption in the early PT would result in a more positive lumen, which in turn would favour retention of chloride and result in increased distal delivery of chloride. This would stimulate tubuloglomerular feedback, thereby decreasing GFR ^97^. The PTH effect on GFR would not directly affect PT calcium transport. However, it would reduce the filtered calcium load and the amount of calcium in the ultrafiltrate. This would decrease the amount of calcium needed to be reabsorbed by active calcium transport in the distal nephron, thereby maximizing calcium reabsorption. Further studies are required to confirm this hypothesis.

The effect of calciophosphotropic hormones on ion transport in the cortical TAL ^98– 100^ and the distal convolution ^67– 69, 71, 101^ have been studied. However, there is a paucity of recent studies looking at the PT. In particular, the potential regulation of tight-junction permeability by calciophosphotropic hormones, including PTH, has received little attention. Functional data suggest a relationship between PTH signalling and altered paracellular transport in the PT ^102^. PTH administration to rats acutely decreased paracellular solute reabsorption from the PT ^102^. This is further supported by a microperfusion study that showed reduced water-driven paracellular solute transport (that is, reduced solvent drag) across rabbit PCTs after infusion of cyclic AMP (a downstream second messenger of PTH-PTHR activation) ^103^. Consistent with the abovementioned studies, inhibition of NHE3 in an intestinal cell culture model resulted in increased TER consistent with reduced tight-junction permeability ^104^ as TER across a leaky epithelium is predominantly a reflection of paracellular ion permeability. Together, these studies suggest that PTH inhibits paracellular transport in the PT by decreasing tight-junction permeability, which likely also affects the permeation of calcium, although this has not been specifically tested. We are unaware of attempts to delineate the molecular components involved in regulation of paracellular permeability following PTH application. Further research is required to do so and to assess the effect of PTH on the transcellular calcium absorption pathway in the late PT.

FGF23 also affects PT solute transport by acting on sodium-phosphate cotransporters. However, given the relatively small amount of sodium reabsorbed via this pathway, this primarily decreases phosphate rather than sodium transport and therefore is discussed below. Currently, we are unaware of data demonstrating an effect of FGF23 on transcellular sodium transport or tight-junction permeability in the PT. It should be kept in mind, however, that FGF23 participates in calcium homeostasis through the enhancement of active vitamin D inactivation as well as by enhancing distal tubular calcium reabsorption through TRPV5 ^34^.

There is evidence of calcium sensing by the PT. The CaSR detects elevated serum calcium. It signals through a G _q/11_ protein–coupled pathway inhibiting PTH release from the parathyroid gland and decreases calcium reabsorption from the TAL ^105, 106^. Several studies have reported CaSR expression in the brush border membrane of PT epithelial cells ^107, 108^, but another study contradicts this observation ^100^. A recent study using both monoclonal and polyclonal antibodies against the CaSR found a low level of expression in PT ^109^. Regardless, a functional study using conditionally immortalized PT epithelial cells isolated from the urine of healthy subjects revealed activation of the G _q/11_ pathway with exposure to increased extracellular calcium as well as its allosteric agonist NPS-R568 ^110^. The physiological role of a CaSR in the PT appears to be to antagonize the inhibitory effects of PTH in PT transport processes. In microperfused late PT (S3 region) and OK cells, the addition of the CaSR agonists gadolinium and NPS R467 abolishes the phosphaturic effects of PTH ^108^. Further microperfusion and micropuncture experiments on rat PT demonstrate a link between CaSR activation and NHE3. Increased fluid absorption and intracellular pH were seen in response to high luminal calcium or NPS-R568, an effect that was absent in CaSR knockout animals ^111^. Together, these studies support the presence of a functional calcium-sensing mechanism in the PT, which antagonizes PTHR activation.

### Human diseases with altered proximal tubular calcium transport

Global PT dysfunction results in glycosuria, aminoaciduria, low-molecular-weight proteinuria and renal tubular acidosis. This constellation of symptoms is called the Fanconi syndrome. Perhaps not surprisingly, the Fanconi syndrome often includes alteration in vitamin D metabolism ^112^. Dent’s disease and the oculo-renal syndrome of Lowe’s disease are typically accompanied by hypercalciuria and nephrocalcinosis ^113, 114^. These diseases are the result of mutations in *CLCN5* or *OCRL1* ^115, 116^. The former gene encodes a transmembrane proton-chloride exchanger (also present intracellularly), and the latter a lipid phosphatase involved in the shuttling of lipid between endomembrane compartments ^117, 118^. Why these gene defects result in a PT calcium phenotype is unknown and is an area of research that requires exploring. Given the possible involvement of CLCN5 in luminal chloride and proton balance, it is possible that loss-of-function mutation in the *CLCN5* gene alters the transepithelial electrical gradient in the PT, thus perturbing various solute transport processes ^117^. However, further studies employing cell and animal models of *CLCN5* and *OCRL1* mutations will help delineate the pathophysiological mechanism of these syndromes.

## Phosphate transport in proximal tubule

### Phosphate reabsorption in the proximal tubule

Phosphate is vital to bone mineralization, maintaining cellular energy stores, and to cell signalling. The kidneys are essential to maintaining systemic phosphate levels, as the majority of ingested phosphate is absorbed from the intestine. Less than 1% of the body’s phosphorus exists in a solubilized form as either dihydrogen phosphate (H _2_PO _4_^1−^) or mono-hydrogen phosphate (HPO _4_^2−^), and the pH determines the fraction of each. The remaining fractions are stored either as part of hydroxyapatite in bone (~85%) or intracellularly (~15%) ^119^. In adults, the kidneys filter approximately 200 mmoles of phosphate daily, and about 90% of it is reabsorbed back into the bloodstream. Of this filtered fraction, 90% is reabsorbed from the PT.

**Figure 3.**
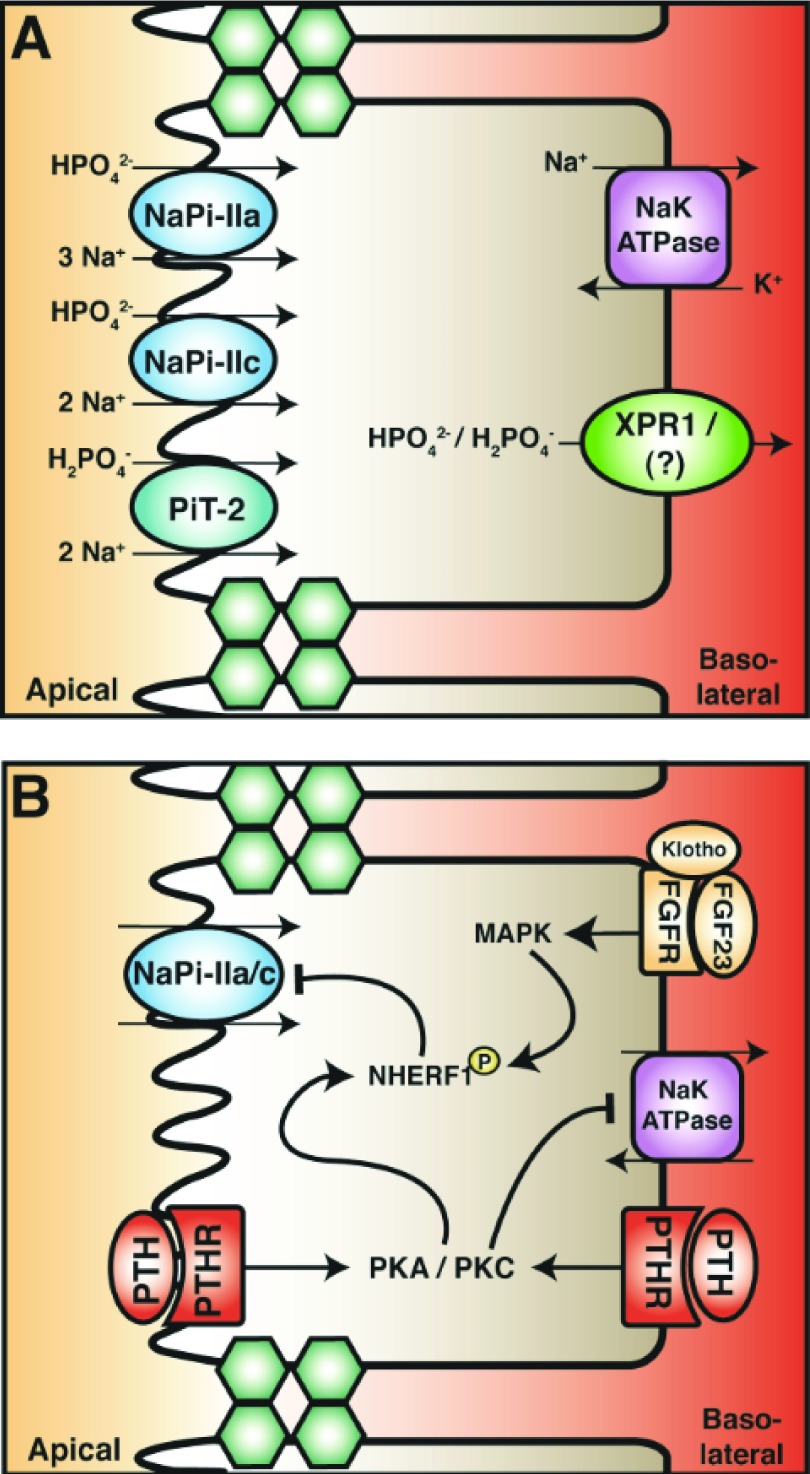
Proximal tubule (PT) phosphate reabsorption. ( **A**) Phosphate reabsorption in the PT is mediated by the transcellular pathway. Apical entry occurs through the sodium-phosphate exchanger family (NaPi) subtypes IIa and IIc and sodium-dependent phosphate transporter 2 (PiT-2). The stoichiometric ratio and preference of phosphate species are depicted. The basolateral extrusion of phosphate may occur through the xenotropic and polytropic retroviral receptor (XPR1). ( **B**) Parathyroid hormone (PTH) and fibroblast growth factor 23 (FGF23) both attenuate phosphate reabsorption in the PT by inhibiting NaPi-II cotransporters. PTH in the tubular fluid and blood activates protein kinase A and C (PKA and PKC). These kinases phosphorylate the PDZ domain–containing scaffold protein sodium hydrogen exchanger regulatory factor 1 (NHERF1), leading to internalization and degradation of NaPi-IIa. FGF23 in the blood binds to its receptor complex (which includes the cofactor, klotho). This leads to the activation of the mitogen-activated protein kinase (MAPK) pathway, resulting in phosphorylation of NHERF1. This signal cascade also decreases NaPi-IIa abundance. How PTH and FGF affect NaPi-IIc is currently unknown.

In the PT, phosphate reabsorption occurs primarily via a transcellular pathway ( Figure 3). Paracellular phosphate reabsorption from this tubule segment has been described as insignificant in comparison with transcellular reabsorption ^4, 5, 120, 121^. Sodium-coupled phosphate cotransporters NaPi-IIa, NaPi-IIc, and PiT-2 mediate the cellular entry of filtered phosphate ions from the lumen ^5^. Apical phosphate entry is facilitated by secondary active transport of sodium, the electrochemical gradient of which is maintained by the basolateral sodium/potassium ATPase. The transport capacity of the PT for phosphate is determined primarily by the abundance of sodium-coupled phosphate transporters, which is due to the steep electrical gradient across the apical membrane (about −70 mV) and a low cytosolic sodium concentration. Expression studies in rodent PT reveal that NaPi-IIa expression is high in the early PT and decreases along the length of this nephron segment, but the fact that NaPi-IIc and PiT-2 are expressed throughout the PT highlights the importance of the early PT to phosphate reabsorption ^122, 123^. The NaPi-II transporter family shows preference for divalent phosphate (HPO _4_^2−^). NaPi-IIa is electrogenic (couples 3 Na ^+^ to 1 phosphate), and NaPi-IIc is electroneutral (couples 2 Na ^+^ to 1 phosphate) ^124– 126^. In contrast, PiT-2 has greater affinity for monovalent phosphate ions (H _2_PO _4_^−^) and is electrogenic ^127, 128^. Genetic knockout studies in mice demonstrate that NaPi-IIa constitutes about 70% of phosphate reabsorption in the PT in this species ^129– 131^. Loss-of-function mutations in the NaPi-IIa cotransporter ( *SLC34A1*) in humans, in contrast to rodents, causes renal calcification and generalized proximal-tubular dysfunction (that is, the Fanconi syndrome) rather than specific phosphate disturbances ^132, 133^. Moreover, NaPi-IIc in the human kidney likely contributes substantially to phosphate reabsorption, as patients with hereditary hypophosphatemic rickets with hypercalciuria—genetic mutations in the NaPi-IIc ( *SLC34A3* gene)—show renal wasting of phosphate due to impaired NaPi-IIc function ^134, 135^. After apical entry, subsequent intracellular diffusion and basolateral extrusion of phosphate complete reabsorption across PT epithelia ^122^. Although little is known about the basolateral extrusion mechanism, a recent nephron-specific knockout of the xenotropic and polytropic retroviral receptor gene ( *Xpr1*) in mice resulted in hypophosphatemia and hyperphosphaturia, suggesting a role for this transporter in renal tubular phosphate reabsorption ^136^. The protein product of *Xpr1* also shares a sequence homology similar to that of a phosphate extrusion transporter in plants (PHO1). Additional experiments need to be carried out to confirm its role in transcellular phosphate reabsorption.

### Regulation of phosphate transport in the proximal tubules

PTH and FGF23 regulate phosphate reabsorption in the PT, which in turn regulates plasma phosphate levels. Active vitamin D increases serum phosphate via enhanced intestinal absorption and potentially via increased PT reabsorption ^137, 138^. However, limited direct evidence of the effect on the PT and confounding effects of PTH and FGF23 complicates this interpretation ^1^. PTH attenuates renal phosphate reabsorption by reducing the membrane abundance of NaPi-IIa, NaPi-IIc, and PiT-2 cotransporters ( Figure 3) ^139– 141^. PTH acutely decreases the abundance of apical NaPi-IIa cotransporters by stimulating endocytosis and ultimately their degradation ^142, 143^. PTH induces NaPi-IIa endocytosis through a complex intracellular pathway, which has been reviewed previously ^4, 5^. In short, the PTH-PTH1R interaction results in phosphorylation of PDZ domain–containing proteins—including NHERF-1—via activation of PKA and PKC, the signalling pathways that inhibit NHE3. NHERF-1 anchors NaPi-IIa to the cytoskeleton and its phosphorylation releases the transporter, permitting endocytosis and degradation in response to PTH ^144– 147^. Patients with mutations in NHERF-1 ( *SLC9A3R1*) display phosphaturia and nephrolithiasis but have otherwise normal PT function ^148, 149^. Interestingly, the mutations are not in the PDZ domain. Instead, these NHERF-1 mutants when expressed *in vitro* confer enhanced PTH-induced cAMP generation and inhibit phosphate transport, suggesting that NHERF-1 is a key component in PTH-mediated phosphaturic effects ^149^. PTH is also implicated in the internalization of NaPi-IIc; however, it is not subsequently degraded ^140, 141^. The molecular pathway whereby NaPi-IIc is downregulated is currently unknown. Nevertheless, like its effect on calcium handling, PTH decreases phosphate reabsorption in the PT. However, unlike calcium, where the distal tubule compensates for calcium loss in PT, the distal nephron has limited ability to reabsorb phosphate. Consequently, elevated PTH induces hyperphosphaturia and hypophosphatemia—symptoms commonly observed in patients with primary hyperparathyroidism ^150^.

Osteocytes and osteoblasts produce FGF23 in response to an increase in plasma phosphate levels and in response to active vitamin D ( Figure 1). FGF23 decreases serum phosphate levels, primarily by reducing phosphate reabsorption from the PT and by reducing intestinal phosphate reabsorption through the inactivation of active vitamin D. In the kidney, FGF23 stimulates the internalization and subsequent degradation of NaPi-IIa and NaPi-IIc cotransporters by phosphorylation of NHERF-1 in a process similar to PTH ( Figure 3B) ^33, 151, 152^. This occurs through mitogen-activated protein kinase (MAPK) and serum/glucocorticoid-regulated kinase-1 (SGK-1) signalling pathways that are activated by FGFR 1, 3, and 4 ^153– 157^. Unlike PTH, the FGF23-FGFR signalling pathway also downregulates transcription and translation of NaPi-IIa and NaPi-IIc cotransporters, contributing to a decrease in abundance of proteins in the PT ^33, 158, 159^. Moreover, PTH-induced endocytosis of NaPi-IIa is abolished by inhibition of the MAPK pathway, suggesting a functional crosstalk mechanism between PTH and FGF23 signalling pathways in the PT ^160^. Thus, the physiological actions of PTH on phosphate excretion and consequent reductions in serum phosphate level are complemented by the action of FGF23.

Direct phosphate sensing is another mechanism by which phosphate transport may be regulated in the PT. A phosphate-sensing mechanism has been observed in cell culture where increased extracellular phosphate activates the MAPK pathway ^161^. This has also been observed in other cell lines, including human embryonic kidney 293 cells ^154, 162^ where increased extracellular phosphate activates the MAPK pathway that FGF23 stimulates, without altering expression of FGF, FGFR, or klotho ^154^. This finding is not surprising when we consider the functional role of FGF23. As a phosphaturic hormone, FGF23 is released in response to high serum phosphate levels. Consequently, FGF23 signals the PT to attenuate the reabsorption of phosphate through NaPi-II cotransporters, inducing phosphate excretion. Therefore, it is likely that increased extracellular phosphate stimulates the same signalling pathway activated by FGF23. However, work remains to confirm this, including exploring the effects of high extracellular phosphate on MAPK signalling *in vivo*, as well as whether phosphate directly regulates gene expression, trafficking, or activity (or a combination of these) of known phosphate transporters in PT.

## Integration of parathyroid hormone and fibroblast growth factor 23 signalling in the proximal tubule

It is evident that PTH and FGF23 have distinct effects in the PT. Both PTH and FGF23 decrease phosphate reabsorption. The mechanism by which PTH and FGF23 attenuate phosphate reabsorption is similar. Both lead to phosphorylation of NHERF-1, resulting in internalization and degradation of NaPi-IIa ^33, 145– 147, 152^. This raises the possibility that there is molecular crosstalk between the PTH and FGF23 signalling pathways ^4, 163^. Although PKA and PKC seem to be the predominant signalling mechanisms for PTH, they also activate the MAPK pathway, which is activated by FGF23 binding the FGFR ^160, 164, 165^. Interestingly, the downstream effects of PTH (that is, internalization of NaPi-IIa) were only partially abolished by PKA and PKC inhibition, but inhibition of MAPK completely abolished NaPi-IIa internalization ^142^. This observation suggests a molecular connection between the PTH and FGF23 pathway, whereby the effect of PTH is dependent on MAPK activation. A recent *in vivo* study by Andrukhova *et al*. revealed that, in mice without FGF23 and klotho, chronic PTH effects are blunted in the PT, an effect restored by recombinant FGF23 administration, further supporting the idea that the actions of PTH are dependent on FGF23 ^3^. This led to the speculation that FGF23 signalling results in the phosphorylation of specific sites on NHERF-1, which are not phosphorylated by PKA or PKC (that is, the downstream mediators of PTH-PTHR). At a systemic level, a similar relationship was observed. FGF23 knockout mice have normal serum PTH but display hyperphosphatemia, consistent with the phosphaturic effect of PTH being dependent on the presence of FGF23 ^158^. Conversely, parathyroidectomized rats, when exposed to active vitamin D which stimulates FGF23 release, do not significantly increase their fractional excretion of phosphate compared with controls, consistent with the FGF23 effect being dependent on PTH ^166^. Similar effects are observed in hypoparathyroid patients who have high serum FGF23 and phosphate levels ^167^. Together, these studies suggest that there is molecular crosstalk between PTHR signalling and FGFR signalling, whereby the phosphaturic effect of PTH and FGF23 is dependent on the other hormone. Overall, PTH and FGF regulation of phosphate balance is a complex process, and much remains to be answered: for example, the molecular mechanism of interaction between second messengers, and the presence of possible reciprocal regulatory mechanisms; that is, does FGF23 signalling activate PKA and PKC?

## Conclusions

PTH and FGF23 are important physiological regulators of calcium and phosphate balance. Calcium reabsorption in the PT occurs primarily by the paracellular pathway, whereas phosphate reabsorption occurs through a transcellular pathway. Reabsorption of both minerals is coupled to sodium. Emerging work has implicated PTH in the direct inhibition of transcellular sodium transport and modulation of the paracellular pathway through which calcium is reabsorbed. Consequently, PTH inhibits calcium reabsorption from the PT, increasing distal delivery, but overall decreases urinary calcium excretion by increasing calcium reclamation from the distal nephron. PTH and FGF23 directly inhibit transcellular phosphate transport in the PT, resulting in increased phosphate excretion. Significant further experimental work is required to fully elucidate the complex PTH-active vitamin D–FGF23 axes in regulating calcium and phosphate transport across the nephron. Of concern, many experimental models are limited by the confounding effects of individual hormones and crosstalk between them. Consistent with this, it seems an unlikely coincidence that both PTH and FGF23 regulate active vitamin D levels via their effect on the PT. Furthermore, whether PTH, active vitamin D, or FGF23 has an effect on PT transcellular calcium transport is not known. Thus, further delineating the molecular pathways mediating calcium and phosphate transport across the PT in the presence and absence of these hormones will contribute to our understanding of renal regulation of calcium and phosphate in both health and disease.
